# A prospective case-control study comparing optical coherence tomography characteristics in neuromyelitis optica spectrum disorder- optic neuritis and idiopathic optic neuritis

**DOI:** 10.1186/s12886-018-0902-3

**Published:** 2018-09-14

**Authors:** Xiujuan Zhao, Wei Qiu, Yuxin Zhang, Yan Luo, Xiulan Zhang, Lin Lu, Hui Yang

**Affiliations:** 1 State Key Laboratory of Ophthalmology, Zhongshan Ophthalmic Center, Sun Yat-Sen University, No. 54 Xianlie South Road, Guangzhou, 510060 People’s Republic of China; 2 Department of Neurology, The Third Affiliated Hospital of Sun Yat-Sen University, Guangzhou, China

**Keywords:** Optical coherence tomography, Optic neuritis, Retinal nerve fiber layer, Neuromyelitis optica

## Abstract

**Background:**

Neuromyelitis optica spectrum disorder-optic neuritis (NMOSD-ON) can now be distinguished from other types of ON as a specific disease by the Aquaporin-4 antibody (AQP4-Ab) test. NMOSD-ON can cause severe retinal nerve fiber layer (RNFL) damage. The optical coherence tomography (OCT) characteristics between NMOSD- ON and idiopathic optic neuritis (IDON) were seldom studied in Asians.

**Methods:**

This prospective case-control study involved 152 eyes from 143 optic neuritis (ON) patients. All the patients were divided into either the NMOSD-ON group or the IDON group based on the AQP4-Ab test. The retinal nerve fiber layer thickness (RNFLT), retinal thickness (RT), and choroidal thickness (CT) were measured by spectral-domain OCT and compared to the 60 age- and gender-matched healthy controls. The association between RNFLT and best corrected visual acuity (BCVA) was examined.

**Results:**

The RNFLT was significantly thinner in all ON patients than in healthy controls, while NMOSD-ON eyes were significantly more affected than IDON eyes in all quadrants (*p* < 0.01). NMOSD-ON patients had stronger visual function impairment than IDON patients (*p* < 0.01). RNFLT was related to BCVA in both the NMOSD-ON and IDON groups. Microcystic macular edema (MME) was identified in 28 patients (19.58%) and in 29 of 152 eyes (19.08%), including 20 of 40 eyes (50%) previously affected by ON. MME was more common in patients with NMOSD-ON (32.2%) than in those with IDON (10.75%) (*p* = 0.001).

**Conclusions:**

The NMOSD-ON group had more pronounced RNFLT thinning and visual function impairment than the IDON group. MME prevalence was higher in NMOSD-ON and was associated with higher frequency of clinical relapses.

## Background

Optic neuritis (ON) is an inflammatory demyelinating disease that involves the optic nerve and causes acute or subacute onset of vision loss [1]. ON is commonly involved in multiple sclerosis (MS), neuromyelitis optica (NMO), and other autoimmune diseases [2]. NMO spectrum disorder-optic neuritis (NMOSD-ON) is one of the common types of ON in Asian. With the finding of aquaporin-4 antibody (AQP4-Ab) [3], NMOSD-ON can now be distinguished from other types of ON as a specific disease [4], as it is present in most patients with NMOSD [5, 6]. What’s more, in 2015, the International Panel for NMO Diagnosis (IPND) achieved consensus that ON with AQP4-Ab seropositivity can be diagnosed as NMOSD [7] and, therefore, ON related to NMOSD was named NMOSD-ON.

Since the definition of NMOSD, NMOSD-ON has been found to differ from other types of ON in many ways. In terms of pathogenesis, NMOSD is characterized by astrocytopathy with demyelination as a secondary involvement, while MS is primarily a demyelinating disease [5]. In laboratory exams, NMOSD differs from MS in serum and cerebrospinal fluid (CSF) examination [8]. For example, NMOSD had more coexisting autoimmunity [9]. Clinically, just as we have reported before [10] and as in many other reports [11, 12] NMOSD-ON has more female preponderance, more bilateral involvement, higher relapse rate, and worse visual prognosis than IDON [6].

Spectral-domain optical coherence tomography (SD-OCT) has been used extensively in ON for quantifying axon damage [13]. Although there have been many reports about OCT results in MS and NMO non-ON eyes [14], the OCT characteristics of ON eyes in Asians are rare. After an acute ON attack in NMOSD-ON and IDON, the OCT features are not so clear. This is especially true for NMOSD-ON, because the concept of NMOSD was only defined recently. As OCT improves in data acquisition speed, resolution and reproducibility [15], different layers in the retina as well as some subtle abnormalities, such as microcystic macular edema (MME), can be more clearly recognized. With the development of enhanced depth imaging (EDI) mode scan in OCT, choroidal thickness (CT) can be accurately measured. All of these advancements broaden the characteristics that can be compared between NMOSD-ON and IDON and improve our understanding about the differences between them. Ethnicity is significant for different types of ON. In Asian populations, the incidence of NMOSD-ON is much higher than reports from Caucasian populations [10]. Although there have been numerous studies about thinner retinal nerve fiber layer thickness (RNFLT), there was a higher incidence of MME in NMO than in MS [16, 17]. Due to the restricted availability of the AQP4-Ab test, research about the OCT characteristics of NMOSD-ON in Asian populations was rare.

To understand the influence of an acute ON attack on the retina and choroid, we analyzed the OCT characteristics six months after acute attacks in a Chinese cohort with NMOSD-ON or IDON, along with healthy controls. The OCT changes of the optic nerve and retinal and CT were then analyzed.

## Methods

Patients with ON were recruited from the neuro-ophthalmology department of Zhongshan Ophthalmic Center. Recruitment took place from November 2013 to July 2015, and patients meeting the inclusion criteria in accordance with the optic neuritis treatment trial (ONTT) [18] with first or relapsing ON were offered participation in this study. Exclusion criteria included any of the following: any evidence of toxic, vascular, infiltrative, compressive, metabolic, hereditary optic neuropathy, causative ocular diseases or retinal lesions [18], fulfilled the diagnostic criteria of MS McDonald’s criteria [19].

Exclusion criteria also comprised an intraocular pressure higher than 21 mmHg, prior ocular trauma, systemic conditions that could affect the visual function, a significant refractive error more than 3D of spherical equivalent refraction or 2D of astigmatism, a history of glaucoma, retinal disease, laser therapy or media opacification. The data were analyzed after the patients had an episode of ON more than six months when the visual functional and structural changes were stabilized. Exclusion criteria also applied to the controls as for ON patients.

The patients were divided into three groups: NMOSD-ON, IDON, and healthy controls. NMOSD-ON included patients who met the established diagnostic criteria for NMO or NMOSD published by Wingerchuk et al. [7]. IDON group patients included those with typical acute demyelinating ON and AQP4-Ab seronegative, none of patients with AQP4-ab negative fullfiled NMOSD criteria or McDonald MS criteria [19].

This study complied with informed consent regulations and the Declaration of Helsinki. A verbal informed consent was needed before subject enrollment in the study.

Laboratory and radiological results were recorded. Serum was drawn for extractable nuclear antigen antibodies (SSA/SSB), antinuclear antibody (ANA), rheumatoid factor (RF), anti-double standard deoxyribonucleic acid (anti-ds DNA), anti-cardiolipin antibodies (ACLs), and AQP4-Ab at the Third Affiliated Hospital of Sun Yat-sen University. All serum samples were analyzed for the presence of AQP4-Ab by indirect immunofluorescence using human AQP4-transfected cells from a commercial BIOCHIP kit (Euroimmun, Germany) as described previously [20]. Clinical data was recorded along with the AQP4-Ab. Patients were sub-divided into the NMOSD-ON group or the IDON group according to the results of the AQP4-Ab test. All patients were treated with corticosteroids.

**Fig. 1:**
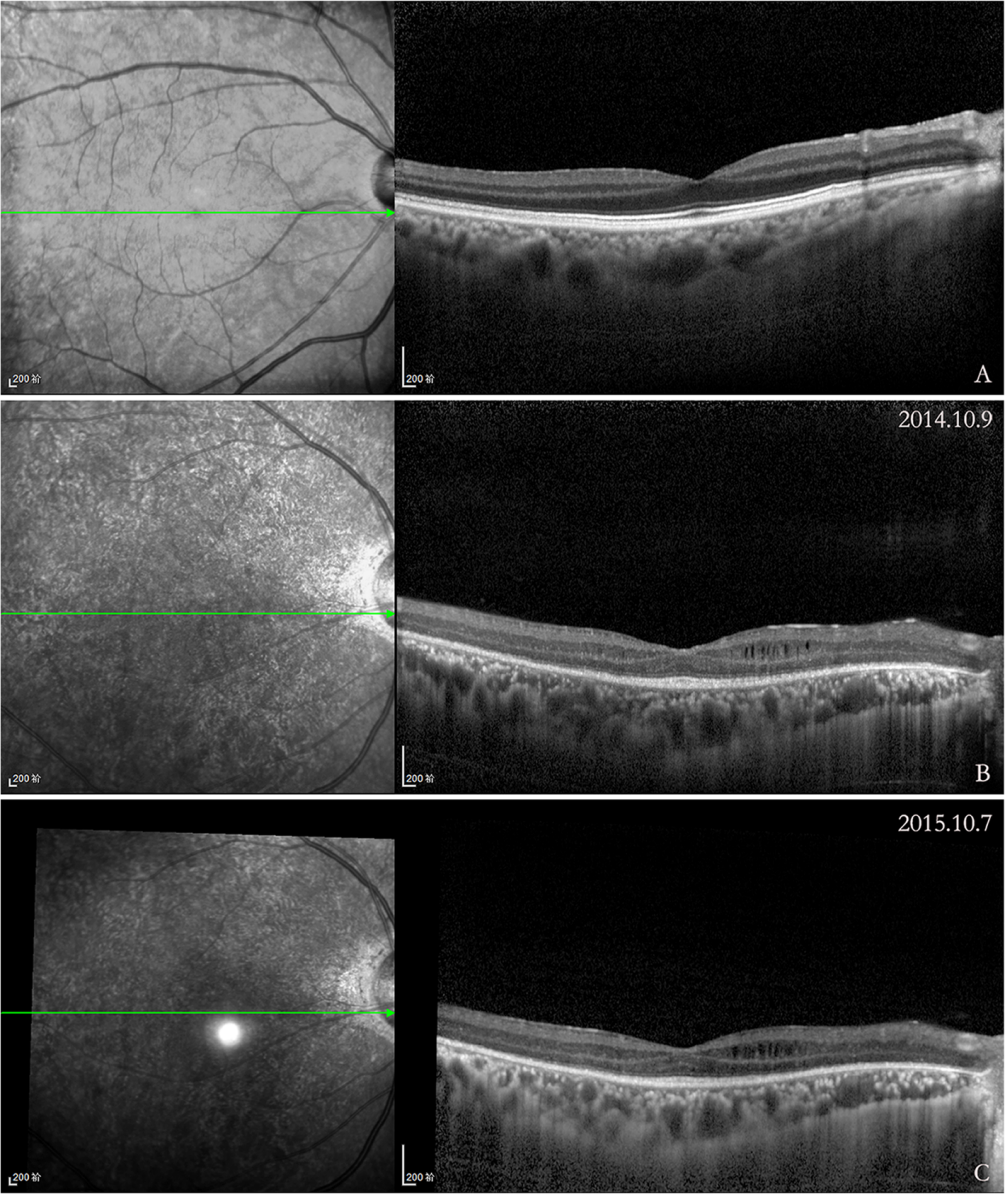
Scans of a NMOSD-ON eyes with and without microcystic macular edema (MME). On the left, it is the infrared fundus image and the green line marks the position of the corresponding OCT image on the right. **a**, Macular scan of NMOSD-ON eye. **b**, MME of the inner nuclear layer on OCT in NMOSD-ON eye. **c**. At one year follow-up, the MME was increased (white arrow)

RNFLT was obtained on a high-definition spectral-domain optical coherence tomography (HD-OCT) with EDI mode (Heidelberg Engineering, Heidelberg, Germany). Measurements were taken using the Spectralis 3.5 mm standard circle scan protocol with version 5.3 software, with signal strength greater than 21. The RNFLT around the optic nerve head in a circle with a minimum of 50 automatic real time (ART) and yields a temporal, superior, nasal, inferior and mean overall graph. Nasal-to-temporal RNFL ratio (N/T ratio) were performed in all patients. A macular scan consisting of 25 horizontal scans centered on the fovea was performed. The central foveal thickness values were automatically generated by the imaging software. CT was defined as the distance between the retinal pigment epithelium (RPE) and the outer border of choroid at the subfoveal location. All OCT measurements were manually performed by one observer who was blinded with the clinical diagnosis and was not involved in the data analysis. MME [21–23] was defined as cystic, honeycombed, lacunar area of hyporeflectivity with clear boundaries in two or more consecutive SD-OCT macular raster scan images [24] (Fig. 1). The BCVA was converted to the logarithm of the minimum angle of resolution (logMAR) for statistical analysis.

### Statistics

All of the data was analyzed using SPSS 19.0 (SPSS Inc., Chicago, IL, USA). First, we determined the mean value (presented as mean ± standard deviation) of RNFLT, central foveal thickness, and CT before we used the one-way ANOVA test and Bonferroni adjustment to compare multiple variables between the different groups. Associations between BCVA and RNFLT were evaluated using the univariate linear regression. All *p* values were two-sided and statistical significance was established at *p* < 0.05.

### Outcome measures

The primary outcome measures were RNFLT compared in NMOSD-ON, IDON patients, and healthy controls. Secondary outcome measures included MME, central foveal thickness, CT, correlations between RNFLT and BCVA.

## Results

A total of 143 patients were evaluated; among them, 59 eyes (52 patients) were in the NMOSD-ON group and 93 eyes (91 patients) were in the IDON group. All NMOSD-ON patients were AQP4-Ab positive. The demographic data of the study cohort is summarized in Table 1.

**Table 1:** Demographic data of NMOSD-ON and IDON patients

Baseline characteristics	NMOSD-ON (*n* = 52)	IDON (*n* = 91)	*P*
Age at first clinical attack, y, median (IQR)	35.00 (20.75–47.00)	37.00 (27.00–49.00)	0.427^a^
Age at serum sampling, y, median (IQR)	36.50 (21.00–47.00)	37.00 (28.00–50.00)	0.196^a^
Sex, F:M	23:3	50:41	0.000^b^
No. of patients with recurrent ON	26	14	0.000^b^
No. of patients with ION	26	77	0.000^b^
No. of ON episodes, mean ± SD	1.58 ± 1.54	1.04 ± 0.19	0.007^a^
Interval relapsing time, m, median (IQR)	12.00 (6.00–48.00)	36.00 (20.25–63.00)	0.237^a^
Interval from onset of last ON attack to OCT test, m, mean ± SD	6.72 ± 1.32	6.31 ± 1.43	0.531^a^
Visual acuity at baseline (logMAR), mean ± SD	2.17 ± 1.38	1.49 ± 1.23	0.044^a^
ON event with visual acuity worse than 1.0 logMAR (n, %)	43, 82.69%	49, 53.85%	0.001^b^
At least one episode with no light perception (n, %)	13, 25.00%	8, 8.79%	0.008^b^
Abnormal brain MRI (n, %)	9, 17.31%	5, 5.49%	0.027^b^
Blood test	37	53	0.50^c^
SSA (n, %)	3, 8.11%	3, 5.66%	
Anti-ds DNA (n, %)	3, 8.11%	1, 1.89%	
ACL (n, %)	1, 2.70%	1, 1.89%	
Accelerated ESR (n, %)	3, 8.11%	1, 1.89%	
Increased CRP (n, %)	2, 5.41%	7, 13.21%	
ANCA positive (n, %)	2, 5.41%	2, 3.77%	
ANA (≥1:320, %)	9, 24.32%	6, 11.32%	
Follow-up outcomes			
No. of cases	48	85	
Visual acuity at last follow-up (logMAR), mean ± SD	1.35 ± 1.30	0.77 ± 0.97	0.001^a^
Cases with myelitis episodes (n, %)	12, 23.08%	1, 1.10%	0.000^b^

*NMOSD-ON* neuromyelitis optica spectrum disorder- optic neuritis, *IDON* idiopathic optic neuritis, *IQR* interquartile range, *ION* isolated optic neuritis, *SSA* anti-Ro/SSA antibody, anti-ds *DNA* anti-double standard deoxyribonucleic acid, *ACL* anti-cardiolipin antibody, *ESR* erythrocyte sedimentation rate, *CRP* C-reactive protein, *ANCA* antineutrophil cytoplasmic antibody, *ANA* antinuclear antibody, ^a^Mann-Whitney U test, ^b^Chi-squared test, ^c^Fisher’s exact test

### OCT measures in ON patients

**Fig. 2:**
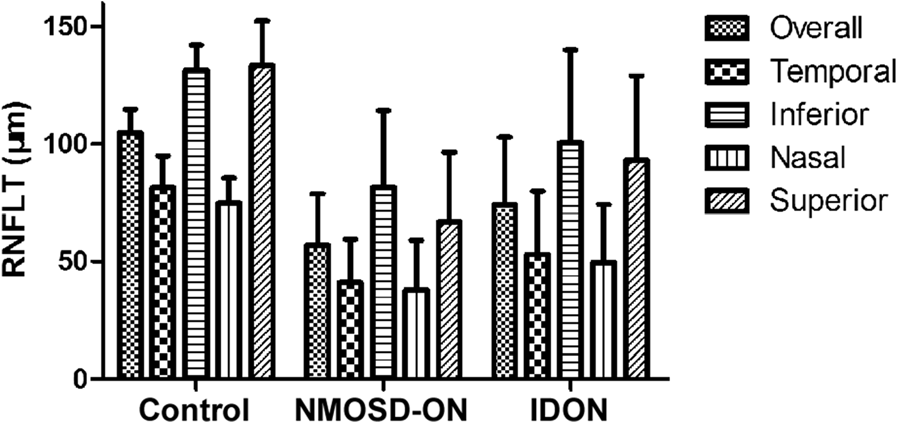
Comparisons of Overall and quadrants RNFLT among control, NMOSD-ON and IDON. Overall and four quadrants RNFLT were significantly thinner in both NMOSD-ON and IDON eyes compared to healthy eyes, with NMOSD-ON eyes being more thinner than IDON eyes significantly (*p* < 0.01). RNFLT: retinal nerve fiber layer thickness; NMOSD-ON: neuromyelitis optica spectrum disorder- optic neuritis; IDON: idiopathic optic neuritis

**Fig. 3:**
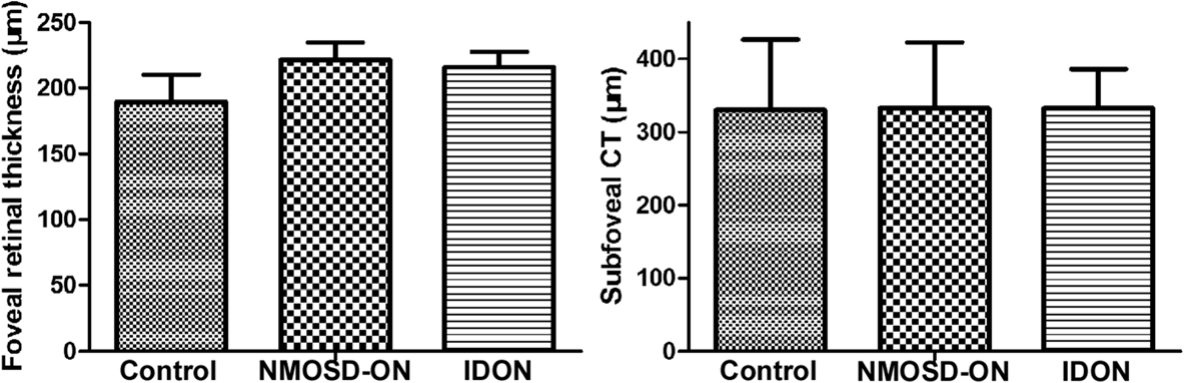
Comparisons of foveal retinal thickness and subfoveal choroidal thickness among control, NMOSD-ON and IDON. Foveal retinal thickness was higher in ON eyes than healthy eyes, and NMOSD-ON eyes higher than IDON eyes (*p* < 0.05). The subfoveal CT in ON eyes was not different from the healthy controls (*p* > 0.05)

Overall RNFLT and four quadrants RNFLT were significantly thinner in both NMOSD-ON and IDON eyes compared to healthy eyes, with NMOSD-ON eyes being more affected than IDON eyes significantly (*p* < 0.01) (Fig. 2). Central foveal thickness trended higher in ON eyes than healthy eyes, and NMOSD-ON eyes higher than IDON eyes (*p* < 0.05). The subfoveal CT in ON eyes was not different from the healthy controls (*p* > 0.05) (Fig. 3). In clinic work, a temporal preponderance was often seen in optic atrophy with ON and OCT showed particular damage to temporal axons [25, 26]. While peripapillary RNFLT (pRNFLT) was primarily thinned in the temporal quadrant in both NMOSD-ON and IDON eyes, pRNFLT of other quadrants in NMOSD-ON eyes was much more reduced than IDON eyes. In order to identify whether a predilection of the temporal quadrant of pRNFL existed, we used the N/T ratio to compare quadrant thinning in the groups of NMOSD-ON and IDON patients [27], but the difference was not statistically significant (0.96 ± 0.43 vs. 1.01 ± 0.46; F = 0.298, *p* = 0.586). NMOSD-ON eyes with a history of one ON event were different from IDON eyes with one ON event (*p* < 0.01). NMOSD-ON eyes with more than one ON event were not different from MS-ON eyes with more than one ON event and NMOSD-ON eyes with one ON event (*p* > 0.05) (Table 2). Both in NMOSD-ON and IDON eyes, BCVA is strongly correlated with overall and four quadrants RNFLT (*p* < 0.05) (Table 3).

**Table 2:** Statistical analysis of overall RNFLT for optic neuritis patients and controls

Overall RNFLT		*P* values
NMOSD-ON with 1 ON (*n* = 32)	Healthy controls (*n* = 60)	0.000
59.67 ± 22.19	105.81 ± 10.60	
IDON with 1 ON (*n* = 79)	Healthy controls (*n* = 60)	0.000
75.48 ± 28.15	105.81 ± 10.60	
NMOSD-ON with 1 ON (*n* = 32)	IDON with 1 ON (*n* = 79)	0.001
59.67 ± 22.19	75.48 ± 28.15	
NMOSD-ON with > 1 ON (*n* = 27)	IDON with > 1 ON (*n* = 14)	0.644
45.44 ± 16.03	51.80 ± 32.51	
NMOSD-ON with 1 ON (*n* = 32)	NMOSD-ON with > 1 ON (*n* = 27)	0.120
59.67 ± 22.19	45.44 ± 16.03	
IDON with 1 ON (*n* = 79)	IDON with > 1 ON (*n* = 14)	0.038
75.48 ± 28.15	51.80 ± 32.51	

*RNFLT* retinal nerve fiber layer thickness, *ON* optic neuritis, *NMOSD-ON* neuromyelitis optica spectrum disorder- optic neuritis, *IDON* idiopathic optic neuritis. Wilcoxon Rank Sum Tests with Holm Correction

**Table 3:** Correlation between BCVA and RNFLT using univariate linear regression

	NMOSD-ON		IDON	
RNFLT	BCVA		BCVA	
	*ß*	*P*	*ß*	*P*
Global	−4.824	0.031	−2.425	0.042
Superior	−7.769	0.026	−1.465	0.028
Nasal	−5.173	0.034	−1.376	0.036
Inferior	−8.435	0.031	−1.168	0.033
Temporal	−4.907	0.044	−2.084	0.047

*NMOSD-ON* neuromyelitis optica spectrum disorder- optic neuritis, *IDON* idiopathic optic neuritis, *BCVA* best corrected visual acuity, *RNFLT* retinal nerve fiber layer thickness

### Role of microcystic macular edema (MME)

MME was identified on OCT in 28 patients (19.58%), and in 29 of 152 eyes (19.08%), including 20 of 40 eyes (50%) with more than one episode of ON. MME was more common in patients with NMOSD-ON (32.20%, 19 of 59 eyes) than in those with IDON (10.75%, 10 of 93 eyes) (*p* = 0.001). A total of 69.0% of eyes with MME had prior ON. There were no appreciable differences in age or sex between patients with and without MME. Eyes with MME had lower vision, but the difference did not reach statistical significance. The overall RNFLT was 19.23 μm thinner in eyes with MME compared to all ON eyes without MME (*p* = 0.03). The overall RNFLT was 5.39 μm lower in eyes with MME compared to ON eyes with prior ON without MME (*p* = 0.36). Central foveal thickness was 10.42 μm higher in eyes with MME compared with eyes without MME (*p* = 0.18).

## Discussion

In this study, a prospective case-control study was made to compare the OCT characteristics between NMOSD-ON and IDON. The study had a large NMOSD-ON sample size and the comparison was not limited to RNFLT, but also included macular and choroidal thicknesses.

As for the clinical features of these patients, there was a strong female predominance, higher recurrent ON frequency, and more severe visual function damage in the NMOSD-ON group.

RNFLT was significantly thinner in NMOSD-ON and IDON patients six months after an ON attack compared to normal controls. It was reported that NMOSD-ON had no preponderant RNFLT thinning pattern [28]. In this study, no significant N/T ratio difference was found in NMOSD-ON versus IDON eyes, which means all quadrants were evenly affected in both types of ON. This was consistent with previous studies from the Asian cohort, which also failed to demonstrate the similar pattern [29]. On the other hand, the severity of the ON studied might also influence the RNFLT pattern. The temporal RNFLT contains the papillomacular bundle, which is more vulnerable to pathologic damage. When the ON attack was not as severe, quadrants other than the temporal might be relatively damaged. Further investigation is needed to identify whether there were indeed pattern differences or ethnic differences in the pathologic involvement of the optic nerve and severity of the ON attack.

Damage of the optic nerve from a single ON attack was more severe in NMOSD patients. RNFLT became much thinner in the NMOSD-ON group than IDON group, which was in line with previous OCT studies [30]. What’s more, this study also demonstrated that RNFLT thinning in first-ever ON was significantly more severe in NMOSD-ON eyes than in IDON eyes. Ratchford et al. [31] suggested that RNFLT differences of more than 15 μm between eyes after a first episode of unilateral ON should prompt consideration of an NMOSD-ON. No significant difference in RNFLT thinning could be found between NMOSD-ON eyes with first-ever ON and those with recurrent ON (RON), which indicated that a single NMOSD-ON attack might be severe enough to destroy most of the RNFLT. This also implied that a functional index, such as visual field or visual evoked potentials, would be more suitable for evaluating the severity of visual damage in RON than RNFLT.

BCVA is strongly correlated with RNFLT, not only with average RNFLT, but also with all quadrants of RNFLT in both NMOSD-ON and IDON eyes. This is in accordance with values reported by earlier studies [29, 32]. Schneider, E., et al. [27] reported that NMOSD-ON eyes showed more apparent association of structural retinal damage and impairment of visual function than in MS-ON eyes. The possible reason may be that half of the NMOSD-ON eyes had pRNFLT below 46.6 μm versus none of the IDON eyes in their study. In our study, there were 19 NMOSD-ON eyes and 17 IDON eyes with pRNFLT below 47 μm. The thinner the RNFLT becomes, the stronger the association between morphology and BCVA becomes. We suggest this might be due to the fact that visual function is no longer able to be maintained after RNFLT decreases to a certain threshold [29, 31].

As for retinal structures, the central foveal thicknesses of both groups were thicker than healthy controls, and NMOSD-ON eyes were thicker than IDON eyes. The differences were primarily driven by MME, which caused a thickening of the inner nuclear layer and outer retinal layers [27]. MME could be a manifestation of inner nuclear layer (INL) pathology. Both Sotirchos et al. [17] and Gelfand et al. [24] reported MME existed in NMO eyes affected by ON. Also, it can be seen in 5–6% of MS patients [6]. MME was associated with more severe MS and poorer VA [33]. In this study, MME was found in 32.2% of NMOSD-ON patients and 10.75% of IDON patients, but not in the healthy control. We also found that MME was associated with more severe RNFLT thinning and more profoundly impaired BCVA. MME occurred in various neuroinflammatory disorders associated with ON [34]. MME might be linked to Müller cell pathology [35], which is caused by AQP4-Ab via a leaky blood-retina barrier. Also, there might be a pathophysiological correlation between the extent of damage to the optic nerve and MME. The findings in this study showed that eyes with more severe ON had a higher incidence of MME.

CT is an index of eye circulation. The concentration of AQP4-Ab in serum was much higher than that in CSF [36–38] and there were reports of retina blood vessel abnormality in NMO [39]. All of these indicating mechanisms of retina blood barrier (RBB) and blood brain barrier (BBB) damage might be involved in the pathogenesis of NMO. Whether different types of ON have an effect on choroidal vessel structure has not yet been studied. Ebru Esen et al. [40] reported that the mean subfoveal CT was reduced significantly in MS patients versus the healthy controls. However, in this study, there was no significant difference between IDON and the normal controls, or between NMOSD-ON and IDON. Diurnal variations of CT [41] could have an effect on the results, because the data was not obtained at the same time of the day. In our study, the number of subfoveal CTs in the normal controls was in accordance with other reports [42, 43] while CT of the healthy control in Ebru Esen et al.’s study was much thicker [40]. Determining whether choroidal vessels are involved in the pathology of the disease requires a larger cohort and more detailed measurements.

Using OCT for differentiation of IDON from NMOSD-ON has long been desirable. However, so far, OCT alone has not been enough for differentiation, though OCT shows a promising effect in investigating the pathologic difference, managing the severity of ON, and evaluating the therapeutic effect on ON [44]. Still, there were some limitations to this study. The relationship between RNFLT change and visual function remains to be investigated. Multilayer segmentation of greater detail should be analyzed. Anti-myelin oligodendrocyte glycoprotein (MOG) antibodies are frequently associated with the recurrent ON/chronic relapsing inflammatory ON phenotype, which is highly sensitive to even low doses of oral corticosteroids [45]. There is still a debate about whether MOG-Ab positive patients will be considered part of the NMOSD, or rather a distinct disease entity [46]. We did not test the anti-MOG antibody in AQP4-Ab negative patients in our study, which may somewhat skew the results. However, this could provide a direction for future research.

## Conclusion

In summary, patients with NMOSD-ON had more pronounced RNFLT thinning than patients with IDON and was closely associated with visual function impairment.

## Abbreviations

ACLs: Anti-cardiolipin antibodies
ANA: Antinuclear antibody
anti-ds DNA: anti-double standard deoxyribonucleic acid
AQP4-Ab: Aquaporin-4 antibody
BCVA: Best corrected visual acuity
CIS: Clinically isolated syndrome
CSF: Cerebrospinal fluid
CT: Choroidal thickness
EDI: Enhanced depth imaging
IDON: Idiopathic optic neuritis
MME: Microcystic macular edema
MS: Multiple sclerosis
MS-ON: MS-related optic neuritis
NMOSD: Neuromyelitis optica spectrum disorder
NMOSD-ON: NMOSD-related optic neuritis
OCT: Optical coherence tomography
ONTT: Optic neuritis treatment trial
RF: Rheumatoid factor
RNFT: Retinal nerve fiber layer thickness
RPE: Retinal pigment epithelium
